# Risk of SARS-CoV-2 Infection Among Households With Children in France, 2020-2022

**DOI:** 10.1001/jamanetworkopen.2023.34084

**Published:** 2023-09-15

**Authors:** Simon Galmiche, Tiffany Charmet, Arthur Rakover, Laura Schaeffer, Olivia Chény, Cassandre von Platen, Faïza Omar, Christophe David, Alexandra Mailles, Fabrice Carrat, Arnaud Fontanet

**Affiliations:** 1 Emerging Diseases Epidemiology Unit, Institut Pasteur, Université Paris Cité, Paris, France; 2 Sorbonne Université, Ecole Doctorale Pierre Louis de Santé Publique, Paris, France; 3 Center for Translational Research, Institut Pasteur, Université Paris Cité, Paris, France; 4 Department of Public Affairs—Public Statistics, Institut Ipsos, Paris, France; 5 Santé Publique France, Saint-Maurice, France; 6 Sorbonne Université, Inserm, IPLESP, Hôpital Saint-Antoine, AP-HP, Paris, France; 7 Unité PACRI, Conservatoire National des Arts et Métiers, Paris, France

## Abstract

**Importance:**

Understanding the contribution of children to SARS-CoV-2 circulation in households is critical for designing public health policies and mitigation strategies.

**Objective:**

To identify temporal changes in the risk of SARS-CoV-2 infection in people living with children.

**Design, Setting, and Participants:**

This case-control study included online questionnaire responses from French adults between October 2020 and October 2022. Eligible cases were adults with ongoing SARS-CoV-2 infection with an email address on record with the national health insurance system, which centralized all new diagnoses in France. Eligible controls were adults who had never tested positive for SARS-CoV-2 until February 2021, when eligibility was extended to all adults without ongoing SARS-CoV-2 infection.

**Exposure:**

Transmission of SARS-CoV-2 from a child (aged under 18 years) within the household in the descriptive analysis, as reported by the participating case. Sharing household with a child (of any age or broken down by school level) in the case-control analysis.

**Main Outcome and Measures:**

Ongoing SARS-CoV-2 infection diagnosed by reverse transcription-polymerase chain reaction or supervised rapid antigen test (ie, not self-tests).

**Results:**

A total of 682 952 cases were included for the descriptive analysis (68.8% female, median [IQR] age, 44 [34-55] years). Among those, 45 108 (6.6%) identified a household child as the source case; this proportion peaked at 10.4% during the Omicron BA.1 wave (December 20, 2021, to April 8, 2022). For the case-control analysis, we matched 175 688 cases (with a 4:1 ratio) for demographic characteristics with 43 922 controls. In multivariable logistic regression analysis, household exposure to children was associated with an increased risk of infection mainly at the end of summer 2021 (receding Delta wave) and during winter 2022 (Omicron BA.1 wave). In subgroup analysis by school level of the child, living with children under the age of 6 was associated with increased odds of infection throughout the study period, peaking at an odds ratio (OR) 1.8 (95% CI, 1.6-2.1) for children looked after by professional in-home caregivers, 1.7 (95% CI, 1.5-1.7) for children in day care facilities, and 1.6 (95% CI, 1.4-1.8) for children in preschool. The ORs associated with household exposure to children aged 6 to 14 years increased during the Delta (August 14, 2021, to December 19, 2021) and Omicron BA.1 waves, reaching 1.6 (95% CI, 1.5-1.7) for primary school children and 1.4 (95% CI, 1.3-1.5) for middle school children. Exposure to older children aged 15 to 17 years was associated with a moderate risk until April 2021, with an OR of 1.2 (95% CI, 1.2-1.3) during curfew in early 2021 (December 4, 2020, to April 8, 2021).

**Conclusions and Relevance:**

The presence of children, notably very young ones, was associated with an increased risk of SARS-CoV-2 infection in other household members, especially during the Delta and Omicron BA.1 waves. These results should help to guide policies targeting children and immunocompromised members of their household.

## Key Points

**Question:**

How did the risk of SARS-CoV-2 infection in people living with children evolve through the pandemic in France?

**Findings:**

In this case-control study, people living with children were at an increased risk of infection. An association with higher risk was consistent through most of the pandemic for households with young children up to 6 years of age, during most of the Delta and Omicron BA.1 waves for households with children of primary school and middle school age, and until vaccination for households with high school students.

**Meaning:**

These results provide guidance for policies focusing on children, particularly very young children, and immunocompromised members of their households.

## Teaser

This case-control study examines the prevalence of SARS-CoV-2 infection in individuals living in households with children over the course of the COVID-19 pandemic in France.

## Introduction

The COVID-19 pandemic toll had reached an estimated 18.2 million excess deaths worldwide by the end of 2021.^1^ France was highly affected with approximately 174 000 confirmed COVID-19 deaths by July 1, 2023.^2^ This stresses the necessity to better understand the main drivers of SARS-CoV-2 transmission, including the contribution of children to transmission within households to assess the relevance of child-targeted interventions, such as vaccination, mask-wearing, ventilation, repeated testing, hybrid education, or school closures.^3,4,5,6^ However, such assessments are difficult, given the large proportion of children with asymptomatic infections who remain undetected.^7,8,9,10,11^

The early evidence suggested that children were less susceptible than adults to infection given lower seroprevalence^12,13^ (1.6% in 0-to-9-year-olds vs above 3% for all other age groups in May 2020 in France^14^). Later assessments, on the other hand, found similar susceptibility based on systematic screening or seroprevalence in settings where in-person schooling had resumed (US, Switzerland, Canada, including as the earlier studies both rural and urban settings).^11,15,16^ The emergence of the more transmissible Delta and Omicron variants might also account for these discrepancies.^17^

Children were also initially found less prone to transmit SARS-CoV-2 in household-based studies: a meta-analysis found SARS infections in households of 4.4% for index cases of children and adolescents aged 0 to 19 years and 10.0% for index cases from individuals aged 20 years or older.^18^ However, after the emergence of variants of concern, children have shown similar SARS infections as adults: the ratio of SARS infections for child index cases vs adult index cases was 0.98 in a meta-analysis.^19^ More detailed analysis of the child’s age found that younger children were more likely than older children to transmit SARS-CoV-2: the risk ratio of secondary transmission from index cases of children aged under 12 years compared with older children was estimated at 1.46 in the same meta-analysis.^19^ This may reflect differences in the contact pattern with other household members depending in the child’s age.

Several studies have reported an increased risk of infection for the members of households with children.^20,21,22,23,24^ Measuring the risk of infection for members of households with children can identify associations regardless of the likelihood of the child being tested and changes in school testing policies, but these associations can evolve following changes in vaccine coverage, the strains circulating in the population, and the interventions focusing on children. We used this approach in the ComCor case-control study,^25,26^ further analyses of which are presented here. We hypothesized that adults sharing a household with children could be exposed to an increased risk of SARS-CoV-2 infection through transmission by the children. Our objective was to assess changes in the associations of the risk of SARS-CoV-2 infection with sharing a household with children of any age in this nationwide case-control study performed among adults in mainland France from October 2020 to October 2022.

## Methods

The design of the ComCor case-control study has been described elsewhere.^25,26^ Cases were identified once weekly through the nationwide information system for positive SARS-CoV-2 tests (reverse transcription-polymerase chain reaction or supervised antigenic rapid diagnostic tests, ie, not self-tests) which was centralized by the national health insurance system (Caisse nationale de l’assurance maladie [CNAM]). After information and online consent, participants completed an online questionnaire on sociodemographic factors, health status, recent activities, and locations visited and provided a description of their household, including whether they lived with a child or a student with optional questions on the school level of that child. Individuals in the case cohort were also asked if they knew how they were infected, who had transmitted SARS-CoV-2 to them, and the age of that person. After screening of the cases’ profiles, a market research company recruited controls (adults who never tested positive for SARS-CoV-2 up to February 2021, without ongoing SARS-CoV-2 infection afterwards) matched via a frequency-matching procedure, for age (ages 18 to 28, 29 to 58, and 59 years and older), sex, region, and the size of the population in the area of residence, in order to limit selection bias. These controls completed the same online questionnaire (except for questions on the circumstances of infection). The participation to the study included this single questionnaire without any follow-up. Further information on study design are available in eMethods in Supplement 1.

This study received ethics approval from the ethics committee Comité de Protection des Personnes Sud Ouest et Outre Mer 1 as required by French regulation on clinical research. The data protection authority, the Commission Nationale de l’Informatique et des Libertés (CNIL), authorized the processing of data on October 21, 2020. Written informed consent was obtained from all participants. This report follows the Strengthening the Reporting of Observational Studies in Epidemiology (STROBE) reporting guideline for observational studies.

We excluded participants with a reported history of infection in the last 2 months (other than the infection for which they were contacted for the study) and those in the case cohort who completed the questionnaire more than 30 days after the onset of symptoms or testing (if asymptomatic) to limit recall errors.

Between October 27, 2020, to October 2, 2022, we investigated temporal changes in the risk of infection associated with living with children, by dividing the study period into 9 shorter periods based on changes in incidence, principal strain in circulation, and major nonpharmaceutical restrictions (eMethods in Supplement 1).

Data on vaccination and incidence in the various age groups were provided by the French government, and by the CNAM for vaccination coverage in 11- to 17-year-olds, as defined on January 1, 2022.^27^ Vaccine coverage remained extremely low for children aged 5 to 10 years, and reached 49% for the second dose in February 2022 for 11-to-14-year-olds and 72% for 15-to-17-year-olds (eMethods in Supplement 1). The proportion of circulating SARS-CoV-2 strains are provided by Santé Publique France and obtained from sequencing studies conducted weekly or fortnightly on a random sample of positive tests.^28^ Data on the demographic structure of the general population in France were obtained from the INSEE (National Institute of Statistics and Economic Studies) online data sets for 2021 (data for people aged 20 years and older were used for approximation of the total adult population).^29^

### Statistical Analysis

We described the proportion of cases reporting transmission from a child aged 17 years or younger living in their household. We also considered the various age categories equivalent to the different school levels (under 3 years for day care or professional in-home caregiver, 3 to 5 years for preschool, 6 to 10 years for primary school, 11 to 14 years for middle school, 15 to 17 years for high school), after the exclusion of cases with inconsistencies between the reported test and symptom onset dates for themselves and the source case.

We analyzed the association between the risk of SARS-CoV-2 infection and living in a household with children by performing an additional exact matching procedure to match more accurately for age (using finer 10-year age categories) and the timing of exposure, as controls were initially enrolled during the week following the inclusion of matched cases. Given the higher number of cases than controls, we matched 4 cases with 1 control for age, sex, region of residence, population size, and calendar week (except for the first period, during which matching was based on whether participants were questioned about exposure before or during the second lockdown). We accounted for the random selection of cases (as there were more than 4 times as many cases than controls) by generating 100 databases of matched sets of 4 cases and 1 control by bootstrapping with replacement, for each period. For each database, we applied a multivariable logistic regression model for the risk of SARS-CoV-2 infection (outcome, ie, case or control status), which included the main exposures of interest, ie, living with a child, either altogether or broken down by school level in a separate model. Beyond matching variables, to account for confounding the model also included sociodemographic characteristics (education level, housing type, number of people in the household, professional category), health status (body mass index, immunosuppression, diabetes, hypertension, chronic respiratory disease), smoking status, COVID-19 vaccination status (number of doses and time since last dose), attendance of private or professional meetings, use of public transportation, travel abroad, sports activities, visits to retail facilities, restaurants, bars, or parties, and health care worker status. Data on race and ethnicity were not available. The choice of the adjusting variables was guided by subject matter knowledge to include all measured causes of the exposure (sharing household with children), the outcome (SARS-CoV-2 infection), or both, relying on the disjunctive cause criterion^30^ (eFigure 1 in Supplement 1). We then extracted the odds ratios (ORs) and the population attributable fraction (PAF) of the 100 models in each period and calculated the median OR and the 2.5th and 97.5th percentiles, for estimation of the 95% confidence intervals. We considered *P* < .05 to be statistically significant and all tests were 2-sided (eMethods in Supplement 1). Data analysis was performed with Stata/SE version 16.0 (StataCorp).

## Results

**Figure 1.**
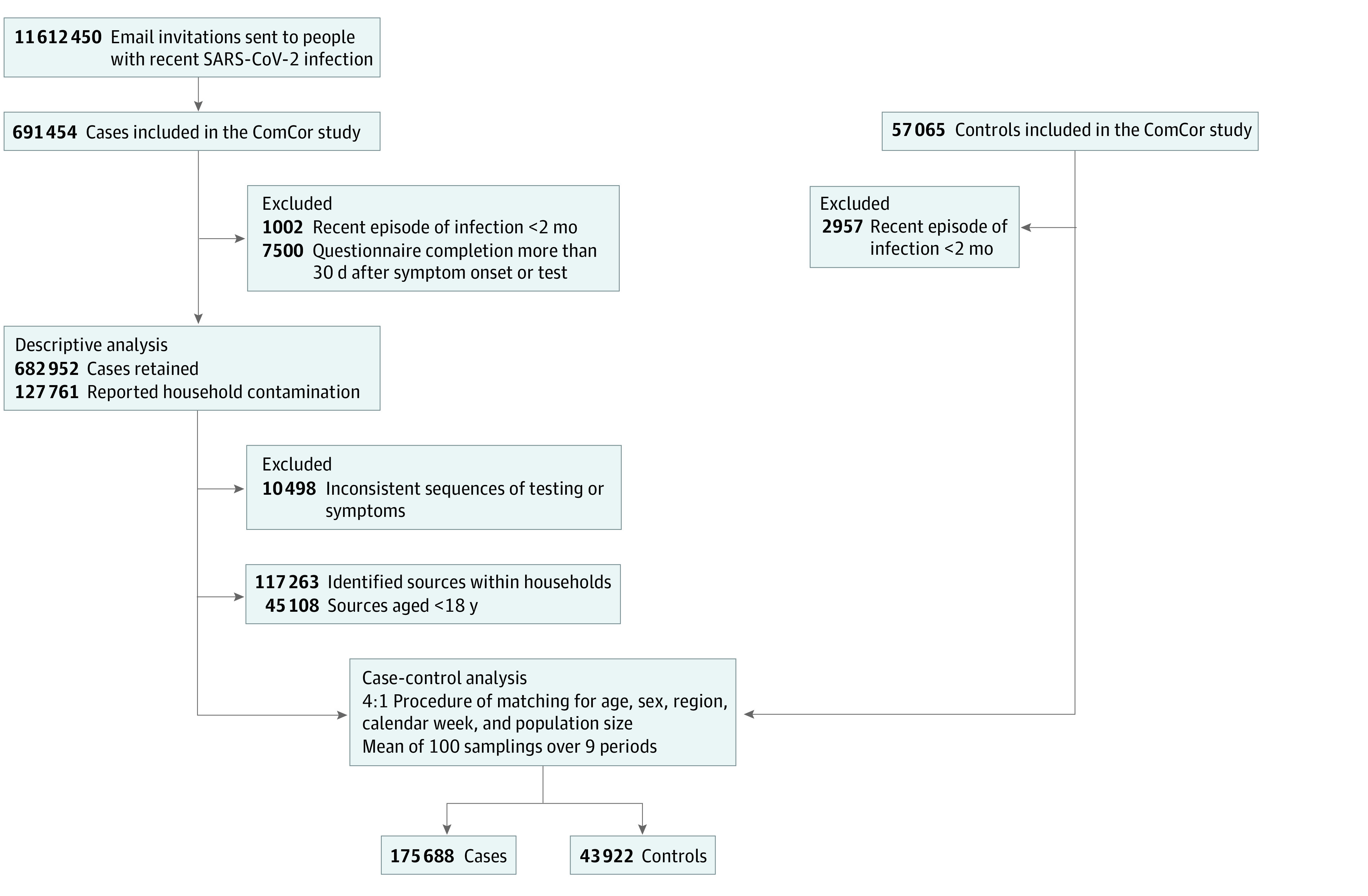
Study Flowchart Descriptive analysis was conducted on cases for which the source case lived in the same household and was aged <18 years. Study conducted in mainland France between October 2020 and October 2022.

From October 27, 2020, to October 2, 2022, 11 612 450 people with recent SARS-CoV-2 infection were invited to participate. A total of 691 454 cases (6.0%) participated in the study, as well as 57 065 controls (Figure 1).

### Description of Intrahousehold Transmissions From a Child

After the exclusion of cases with an inconsistent reported history of SARS-CoV-2 infection and with questionnaires completed more than 30 days after symptom onset or testing, we included 682 952 cases for the descriptive analysis (median [IQR] age, 44 [34-55] years; 469 579 female [68.8%]). In total, 254 401 of these cases (37.3%) were able to identify the source of their contamination, and 117 263 (17.2%) identified someone from their household as the probable source (Figure 1). We identified 45 108 cases (6.6%) for whom the source case was a member of the household under the age of 18 years. This group of cases had a higher frequency of women than the rest of the cases (35 153 [77.9%] vs 434 426 [68.1%]; *P* < .001) and was also younger (median [IQR] age, 41 [37-45] years vs 45 [34-55] years; *P* < .001). The proportion of cases identifying a child as the source case varied over the course of the study (Table 1). It remained below 5% between October 2020 and the summer of 2021 (periods 1 to 4). It then increased to 7.7% in August-September 2021 (period 5, receding Delta wave) and reached a maximum of 10.4% in the winter of 2021-2022 (period 7, Omicron BA.1 wave), before decreasing again to 3.0% in the summer of 2022 (period 9, Omicron BA.4/BA.5 wave). Considerable variations were observed over time for age groups 3 to 5 years, 6 to 10 years, and 11 to 14 years (Table 1). These changes paralleled the increase in the proportion of school-aged children among all cases diagnosed in the fall of 2021 according to the national database (eFigures 2 and 3 in Supplement 1) (eg, children aged 6-10 years accounted for up to 13.7% of all positive tests in France in period 6^27^).

**Table 1.** Proportion of Cases Living in the Same Household as Child or Adolescent Who Was Source Case

Age group of the source case, y	Cases, %									Total
	1	2	3	4	5	6	7	8	9	
0-2	0.1	0.3	0.5	0.2	0.6	0.4	0.8	0.6	0.4	0.5
3-5	0.2	0.5	0.7	0.3	1.3	1.2	2.0	0.9	0.6	1.1
6-10	0.4	1.1	1.3	1.0	3.5	4.8	3.9	2.8	1.1	2.5
11-14	0.7	1.2	1.1	1.1	1.4	1.7	2.2	1.8	0.6	1.5
15-17	0.9	1.1	1.1	1.3	0.9	0.5	1.5	1.0	0.3	1.0
Total	2.4	4.4	4.7	3.9	7.7	8.6	10.4	7.0	3.0	6.6

Analysis of a case-control study conducted in mainland France between October 2020 and October 2022. Individuals in the case cohort were asked if they knew the source of the infection. Study period divided according to changes in incidence, predominant circulating strain, and major nonpharmaceutical interventions. The start dates for the periods were as follows: (1) October 1, 2020; (2) December 4, 2020; (3) April 9, 2021; (4) June 14, 2021; (5) August 14, 2021; (6) October 2, 2021; (7) December 20, 2021; (8) March 18, 2022; (9) May 20, 2022 (study end date: October 2, 2022). No period could be defined for 2309 cases for whom dates of symptom onset and testing were unavailable.

### Case-Control Analysis on the Association of Sharing Household With a Child With the Risk of Infection

After the matching and bootstrapping process, we included 175 688 cases and 43 922 controls (sum of the mean values for the 100 databases for each period) in the case-control analysis for the risk associated with living with a child (Figure 1). The population included in this analysis had a higher proportion of women (66.2% vs 52.4% in the general population aged 20 and over in France), the most frequently represented age group was 40 to 49 years (26.8% vs 16.7%), and a moderately higher proportion of residents of the Ile-de-France region (where Paris is located) (22.1% vs 18.3%) (Table 2). Close to half of all participants reported sharing the household with a child of any age or school level: 82 484 (46.9%) of cases and 19 652 (44.7%) of controls. The most frequently reported school levels were primary school (29 039 cases [16.5%]; 6185 controls [14.1%]) and middle school (26 790 cases [15.2%]; 6183 controls [14.1%]) (Table 2). Further description of the population for the case-control analysis are provided in the supplement (eTable in Supplement 1).

**Table 2.** Study Population Characteristics

Variable	Participants, No. (%)^a^		*P* value
	Cases	Controls	
Total	175 688	43 922	
Sex			
Female	116 308 (66.2)	29 077 (66.2)	>.99
Male	59 380 (33.8)	14 845 (33.8)	
Age, y			
18-29	21 244 (12.1)	5311 (12.1)	>.99
30-39	31 888 (18.2)	7972 (18.2)	
40-49	47 032 (26.8)	11 758 (26.8)	
50-59	37 960 (21.6)	9490 (21.6)	
60-69	24 796 (14.1)	6199 (14.1)	
≥70	12 768 (7.3)	3192 (7.3)	
Population in the area of residence			
<5000 inhabitants	44 828 (25.5)	11 207 (25.5)	>.99
5000-19 999 inhabitants	13 984 (8.0)	3496 (8.0)	
20 000-99 999 inhabitants	17 800 (10.1)	4450 (10.1)	
Over 100 000 inhabitants	61 956 (35.3)	15 489 (35.3)	
Greater Paris area	37 120 (21.1)	9280 (21.1)	
Region of residence			
Ile-de-France	39 848 (22.7)	9962 (22.7)	>.99
Auvergne-Rhône-Alpes	23 384 (13.3)	5846 (13.3)	
Occitanie	17 860 (10.2)	4465 (10.2)	
Provence-Alpes-Côte d’Azur and Corsica	15 788 (9.0)	3947 (9.0)	
Nouvelle-Aquitaine	15 132 (8.6)	3783 (8.6)	
Grand Est	15 336 (8.7)	3834 (8.7)	
Hauts-de-France	14 800 (8.4)	3700 (8.4)	
Pays de la Loire	8580 (4.9)	2145 (4.9)	
Bretagne	8324 (4.7)	2081 (4.7)	
Normandie	6124 (3.5)	1531 (3.5)	
Bourgogne-Franche-Comté	5876 (3.3)	1469 (3.3)	
Centre-Val de Loire	4636 (2.6)	1159 (2.6)	
Education level			
No diploma	3682 (2.1)	767 (1.7)	<.001
Pre–high school diploma	25 982 (14.8)	7722 (17.6)	
High school diploma	31 763 (18.1)	10 559 (24.0)	
Bachelor’s degree	62 869 (35.8)	16 340 (37.2)	
Master’s degree or higher	51 392 (29.3)	8534 (19.4)	
Health care worker	19 400 (11.0)	3021 (6.9)	<.001
Health conditions			
Diabetes	6149 (3.5)	2304 (5.2)	<.001
Hypertension	20 480 (11.7)	5773 (13.1)	<.001
Chronic respiratory disease	14 639 (8.3)	3132 (7.1)	<.001
Body mass index^b^			
Healthy weight (≥18.5 or <25)	89 350 (50.9)	20 909 (47.6)	<.001
Underweight (<18.5)	5414 (3.1)	1880 (4.3)	
Overweight (≥25 &<30)	52 848 (30.1)	13 111 (29.9)	
Obesity (≥30)	28 074 (16)	8022 (18.3)	
Housing			
Individual house	106 790 (60.8)	25 775 (58.7)	<.001
Apartment	68 217 (38.8)	18 004 (41.0)	
Other	681 (0.4)	143 (0.3)	
Sharing household with a child of any age and school level	82 484 (46.9)	19 652 (44.7)	<.001
Sharing household with a child, by school level			
Daycare	4946 (2.8)	928 (2.1)	<.001
Professional in-home caregiver	5779 (3.3)	904 (2.1)	<.001
Preschool	16 478 (9.4)	3327 (7.6)	<.001
Primary school	29 039 (16.5)	6185 (14.1)	<.001
Middle school	26 790 (15.2)	6183 (14.1)	<.001
High school	21 989 (12.5)	5447 (12.4)	.54
Study period (onset date)^c^			
1 (October 1, 2020)	7308 (4.2)	1827 (4.2)	
2 (December 4, 2020)	19 636 (11.2)	4909 (11.2)	
3 (April 9, 2021)	9008 (5.1)	2252 (5.1)	
4 (June 14, 2021)	11 264 (6.4)	2816 (6.4)	
5 (August 14, 2021)	4820 (2.7)	1205 (2.7)	
6 (October 02, 2021)	11 248 (6.4)	2812 (6.4)	
7 (December 20, 2021)	44 136 (25.1)	11 034 (25.1)	
8 (March 18, 2022)	39 652 (22.6)	9913 (22.6)	
9 (May 20, 2022-October 2, 2022)	28 616 (16.3)	7154 (16.3)	

^a^ Cases were adults recently diagnosed with ongoing SARS-CoV-2 infection who were invited by email by the national health insurance system. The controls are adults from the general population matched for age, sex, size of the population in the area of residence, region, and calendar week, who had never tested positive for SARS-CoV-2 until February 2021, when eligibility was extended to all adults without ongoing SARS-CoV-2 infection. One control was matched with 4 cases on age, sex, region, population size of the area of residence, and week of exposure with a bootstrapping procedure to generate 100 databases for each of the study periods to account for the random selection of cases. The numbers indicated are the means for the 100 databases generated for each period by bootstrapping with replacement of sets of 1 control for 4 cases, summed over the 9 periods.

^b^ Calculated as weight in kilograms divided by height in meters squared.

^c^ Study periods based on changes in incidence, the principal circulating strain, and major nonpharmaceutical interventions.

**Figure 2.**
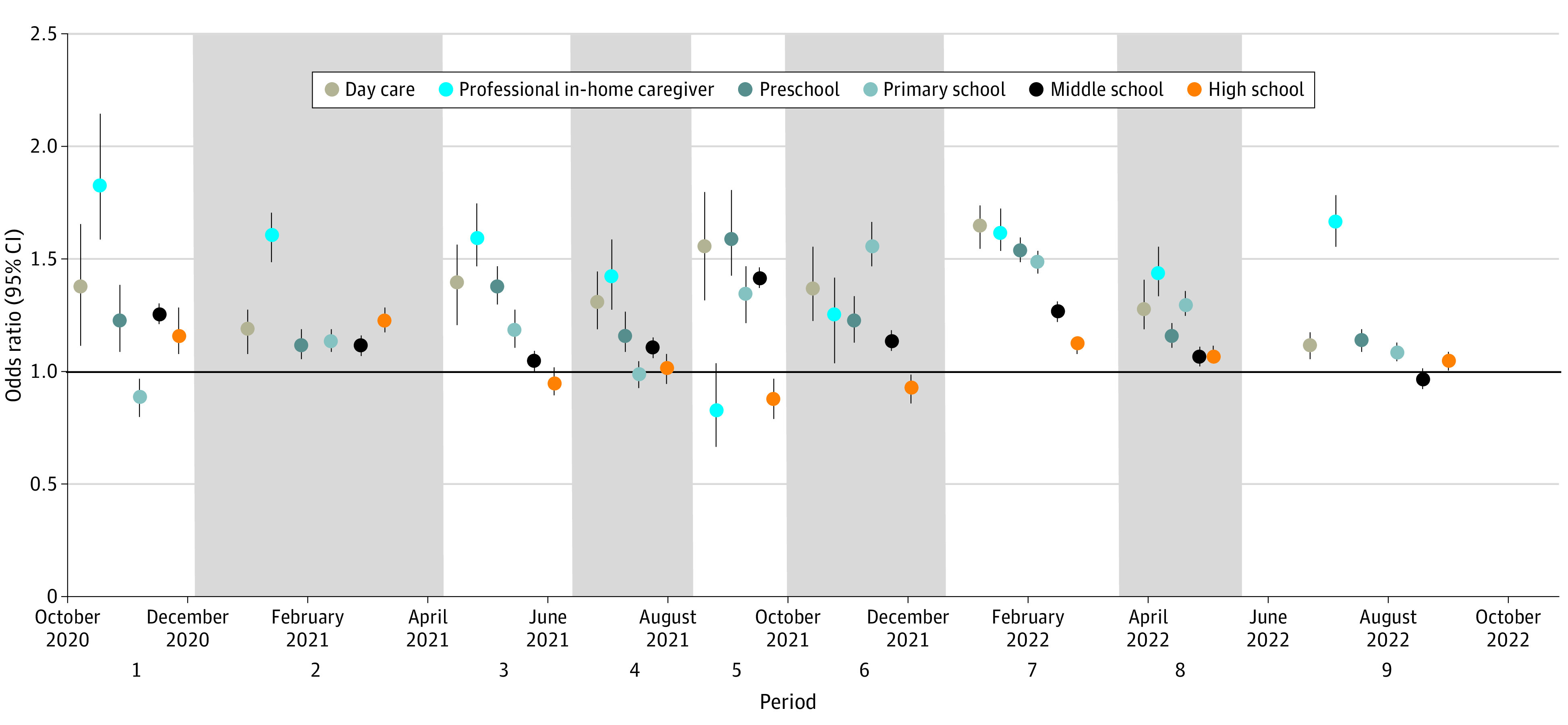
Risk of SARS-CoV-2 Infection in People Living With Children by Age and School Level Odds ratios in fitted multivariable logistic regression models. The periods were defined on the basis of changes in SARS-CoV-2 incidence, predominant circulating strain, and major nonpharmaceutical interventions (the periods are indicated by shaded gray areas), with start dates as follows: (1) October 1, 2020; (2) December 4, 2020; (3) April 9, 2021; (4) June 14, 2021; (5) August 14, 2021; (6) October 2, 2021; (7) December 20, 2021; (8) March 18, 2022; (9) May 20, 2022 (study end date: October 2, 2022). Multivariable logistic regression models are adjusted for sociodemographic characteristics, health status (body mass index, immunosuppression, diabetes, hypertension, chronic respiratory disease), smoking status, COVID-19 vaccination status (number of doses and time since last dose), attendance of private or professional meetings, use of public transportation, travel abroad, sports activities, visits to retail facilities, restaurants, bars, or parties, and health care worker status.

The risk of SARS-CoV-2 infection associated with living with a child changed over the course of the study (Figure 2, Table 3). The largest increases in risk for living with a child of any age or school level were found during the receding of the Delta wave at the end of summer 2021 (period 5: OR, 1.4; 95% CI, 1.3-1.5) and during the Omicron BA.1 wave in the winter of 2022 (period 7: OR, 1.3; 95% CI, 1.3-1.3), whereas there was no increase in risk during periods 1 (second wave; historical strain), 4 (summer 2021; emergence of the Delta variant), and 6 (fall 2021, Delta variant) (Table 3; eFigure 4 in Supplement 1).

**Table 3.** Prevalence of Exposure to a Child in the Household by School Level and Age Group

Households with a child^a^	Period	No. (%)		Odds of infection, OR (95% CI)		Attributable fraction (95% CI)
		Prevalence in cases	Prevalence in controls	Unadjusted	Adjusted	
Any age or school level	1	3678 (50.3)	833 (45.6)	1.2 (1.2-1.3)	0.9 (0.9-1.0)	NA^b^
	2	10 703 (54.5)	2426 (49.4)	1.2 (1.2-1.3)	1.1 (1.1-1.2)	6.9 (4.2-9.2)
	3	5233 (58.1)	1213 (53.9)	1.2 (1.2-1.2)	1.1 (1.1-1.2)	7.8 (4.7-11.5)
	4	5252 (46.6)	1451 (51.6)	0.8 (0.8-0.8)	1.0 (0.9-1.1)	NA^b^
	5	2575 (53.5)	594 (49.3)	1.2 (1.1-1.2)	1.4 (1.3-1.5)	15.8 (11.6-21.4)
	6	5351 (47.9)	1270 (45.2)	1.1 (1.1-1.1)	1.0 (1.0-1.1)	1.1 (NA^b^-3.7)
	7	20 350 (46.1)	4452 (40.4)	1.3 (1.3-1.3)	1.3 (1.3-1.4)	11.4 (10.1-12.8)
	8	16 915 (42.7)	4134 (41.7)	1.0 (1.0-1.1)	1.1 (1.1-1.2)	5.5 (3.8-6.9)
	9	12 427 (43.4)	3278 (45.8)	0.9 (0.9-0.9)	1.1 (1.0-1.1)	3.6 (1.3-4.9)
Attending day care	1	160 (2.2)	27 (1.5)	1.5 (1.3-1.7)	1.4 (1.1-1.7)	0.6 (0.2-1.0)
	2	619 (3.2)	122 (2.5)	1.3 (1.2-1.4)	1.2 (1.1-1.3)	0.5 (0.2-0.7)
	3	329 (3.7)	58 (2.6)	1.4 (1.3-1.6)	1.4 (1.2-1.6)	1.1 (0.6-1.5)
	4	419 (3.7)	90 (3.2)	1.2 (1.1-1.3)	1.3 (1.2-1.4)	0.9 (0.6-1.2)
	5	168 (3.5)	30 (2.5)	1.4 (1.3-1.6)	1.6 (1.3-1.8)	1.2 (0.8-1.7)
	6	294 (2.6)	55 (2.0)	1.3 (1.2-1.5)	1.4 (1.2-1.6)	0.7 (0.5-1.0)
	7	1220 (2.8)	183 (1.7)	1.7 (1.6-1.8)	1.7 (1.5-1.7)	1.1 (0.9-1.2)
	8	965 (2.4)	183 (1.8)	1.3 (1.2-1.4)	1.3 (1.2-1.4)	0.5 (0.4-0.7)
	9	771 (2.7)	180 (2.5)	1.1 (1.0-1.1)	1.1 (1.1-1.2)	0.3 (0.1-0.5)
Looked after by a professional in-home caregiver	1	217 (3.0)	25 (1.4)	2.2 (1.9-2.4)	1.8 (1.6-2.1)	1.4 (1.0-1.7)
	2	795 (4.0)	111 (2.3)	1.8 (1.7-1.9)	1.6 (1.5-1.7)	1.6 (1.2-1.8)
	3	402 (4.5)	58 (2.6)	1.8 (1.7-1.9)	1.6 (1.5-1.7)	1.7 (1.4-2.1)
	4	292 (2.6)	66 (2.3)	1.1 (1.0-1.2)	1.4 (1.3-1.6)	0.8 (0.5-1.0)
	5	102 (2.1)	32 (2.7)	0.8 (0.7-0.9)	0.8 (0.7-1.0)	NA^b^
	6	292 (2.6)	54 (1.9)	1.4 (1.2-1.5)	1.3 (1.0-1.4)	0.5 (0.1-0.8)
	7	1499 (3.4)	214 (1.9)	1.8 (1.7-1.9)	1.6 (1.5-1.7)	1.3 (1.1-1.5)
	8	1263 (3.2)	206 (2.1)	1.5 (1.5-1.6)	1.4 (1.3-1.6)	1.0 (0.8-1.2)
	9	918 (3.2)	138 (1.9)	1.7 (1.6-1.8)	1.7 (1.6-1.8)	1.3 (1.1-1.5)
Attending preschool	1	562 (7.7)	103 (5.6)	1.4 (1.3-1.5)	1.2 (1.1-1.4)	1.4 (0.6-2.3)
	2	2122 (10.8)	435 (8.9)	1.2 (1.2-1.3)	1.1 (1.1-1.2)	1.1 (0.6-1.8)
	3	1203 (13.4)	216 (9.6)	1.5 (1.4-1.5)	1.4 (1.3-1.5)	3.8 (3.0-4.6)
	4	942 (8.4)	227 (8.1)	1.0 (1.0-1.1)	1.2 (1.1-1.3)	1.2 (0.7-1.8)
	5	610 (12.7)	91 (7.6)	1.8 (1.7-1.9)	1.6 (1.4-1.8)	4.8 (3.7-6.1)
	6	1119 (9.9)	218 (7.8)	1.3 (1.2-1.4)	1.2 (1.1-1.3)	1.9 (1.1-2.7)
	7	4441 (10.1)	759 (6.9)	1.5 (1.5-1.6)	1.5 (1.5-1.6)	3.6 (3.3-3.9)
	8	3104 (7.8)	695 (7.0)	1.1 (1.1-1.2)	1.2 (1.1-1.2)	1.1 (0.7-1.5)
	9	2374 (8.3)	583 (8.1)	1.0 (1.0-1.1)	1.1 (1.1-1.2)	1.0 (0.7-1.3)
Attending primary school	1	1103 (15.1)	258 (14.1)	1.1 (1.0-1.1)	0.9 (0.8-1.0)	NA^b^
	2	3632 (18.5)	763 (15.5)	1.2 (1.2-1.3)	1.1 (1.1-1.2)	2.3 (1.5-3.1)
	3	1940 (21.5)	391 (17.4)	1.3 (1.3-1.4)	1.2 (1.1-1.3)	3.6 (2.0-4.9)
	4	1648 (14.6)	451 (16.0)	0.9 (0.9-0.9)	1.0 (0.9-1.0)	NA
	5	1044 (21.7)	197 (16.3)	1.4 (1.3-1.5)	1.4 (1.2-1.5)	5.8 (3.9-7.4)
	6	2293 (20.4)	389 (13.9)	1.6 (1.5-1.6)	1.6 (1.5-1.7)	7.6 (6.6-8.8)
	7	7663 (17.4)	1397 (12.7)	1.4 (1.4-1.5)	1.5 (1.4-1.5)	5.9 (5.4-6.4)
	8	5865 (14.8)	1304 (13.2)	1.1 (1.1-1.2)	1.3 (1.2-1.4)	3.5 (3.0-4.0)
	9	3850 (13.5)	1035 (14.5)	0.9 (0.9-0.9)	1.1 (1.0-1.1)	1.1 (0.6-1.6)
Attending middle school	1	1301 (17.8)	244 (13.4)	1.4 (1.3-1.5)	1.3 (1.2-1.4)	3.8 (2.8-5.2)
	2	3557 (18.1)	755 (15.4)	1.2 (1.2-1.2)	1.1 (1.1-1.2)	2.0 (1.3-2.7)
	3	1750 (19.4)	398 (17.7)	1.1 (1.1-1.2)	1.0 (1.0-1.1)	0.9 (NA^b^-2.0)
	4	1726 (15.3)	422 (15.0)	1.0 (1.0-1.1)	1.1 (1.0-1.2)	1.5 (0.6-2.2)
	5	847 (17.6)	164 (13.6)	1.4 (1.3-1.4)	1.4 (1.3-1.5)	5.4 (4.0-6.5)
	6	1781 (15.8)	391 (13.9)	1.2 (1.1-1.2)	1.1 (1.0-1.2)	1.9 (0.7-3.0)
	7	6796 (15.4)	1404 (12.7)	1.2 (1.2-1.3)	1.3 (1.2-1.3)	3.3 (2.9-3.8)
	8	5433 (13.7)	1365 (13.8)	1.0 (1.0-1.0)	1.1 (1.0-1.1)	0.9 (0.5-1.4)
	9	3600 (12.6)	1040 (14.5)	0.8 (0.8-0.9)	1.0 (0.9-1.0)	NA^b^
Attending high school	1	1252 (17.1)	252 (13.8)	1.3 (1.2-1.4)	1.2 (1.1-1.3)	2.5 (1.2-4.0)
	2	3162 (16.1)	654 (13.3)	1.2 (1.2-1.3)	1.2 (1.2-1.3)	3.1 (2.4-3.8)
	3	1366 (15.2)	353 (15.7)	1.0 (0.9-1.0)	1.0 (0.9-1.0)	NA^b^
	4	1427 (12.7)	381 (13.5)	0.9 (0.9-1.0)	1.0 (1.0-1.1)	0.3 (NA^b^-1.0)
	5	611 (12.7)	163 (13.5)	0.9 (0.9-1.0)	0.9 (0.8-1.0)	NA^b^
	6	1288 (11.4)	355 (12.6)	0.9 (0.9-0.9)	0.9 (0.9-1.0)	NA^b^
	7	5257 (11.9)	1251 (11.3)	1.1 (1.0-1.1)	1.1 (1.1-1.2)	1.3 (0.9-1.7)
	8	4420 (11.1)	1157 (11.7)	1.0 (0.9-1.0)	1.1 (1.0-1.1)	0.8 (0.4-1.2)
	9	3207 (11.2)	881 (12.3)	0.9 (0.9-0.9)	1.0 (1.0-1.1)	0.5 (0.1-0.9)

Abbreviation: NA, not applicable.

^a^ For each school level, the reference category is not living with a child of that same school level. The study period was divided into 9 periods according to changes in incidence, predominant circulating strain, and major nonpharmaceutical interventions. The start dates for the periods were as follows: (1) October 1, 2020; (2) December 4, 2020; (3) April 9, 2021; (4) June 14, 2021; (5) August 14, 2021; (6) October 2, 2021; (7) December 20, 2021; (8) March 18, 2022; (9) May 20, 2022 (study end date: October 2, 2022). Multivariable logistic regression models are adjusted for sociodemographic characteristics, health status (body mass index, immunosuppression, diabetes mellitus, hypertension, chronic respiratory disease), smoking status, COVID-19 vaccination status (number of doses and time since last dose), attendance of private or professional meetings, use of public transport, travel abroad, sports activities, visits to retail facilities, restaurants, bars, or parties, and health care worker status. The choice of the adjusting variables was guided by subject-matter knowledge to include all measured causes of the exposure (sharing household with children), the outcome (SARS-CoV-2 infection), or both.

^b^ Point estimate negative.

When broken down by school level, we identified that people living with children in the youngest age groups (under 6 years of age) had increased odds of infection, particularly for those living with children looked after by a professional in-home caregiver (eg, period 1: OR, 1.8; 95% CI, 1.6- 2.1), and remained relatively stable throughout the study. The proportion of participants with children in these age groups being low, the PAFs remained usually below 2% (Table 3).

People living with children attending primary school (ages 6 to 10 years) initially had lower odds of infection. They increased notably from August to September 2021 (period 5) onwards during the Delta and Omicron BA.1 waves (eg, period 4: OR, 1.0; 95% CI, 0.9-1.0 vs period 5: OR, 1.4; 95% CI, 1.2-1.5). The proportion of people living with children attending primary school being high (approximately 15% of controls), the PAFs obtained for this age group were the highest for any age group during the Delta and Omicron BA.1 waves (period 6: 7.6%; 95% CI, 6.6%-8.8%).

For people living with children attending middle school (ages 11 to 14 years), the increase in risk varied over the study period, with OR point estimates peaking at 1.3-1.4 during the second wave of the historical strain (period 1: OR, 1.3; 95), the receding of the Delta wave at the end of summer 2021 (period 5: OR, 1.4; 95% CI, 1.3-1.5), and the Omicron BA.1 wave in winter 2022 (period 7: OR, 1.3; 95% CI, 1.2-1.3). As the ORs were lower than for children attending primary school, the PAFs were lower for this age group despite similar proportion of population exposed (Table 3). For people living with children attending high school (ages 15 to 17 years), increases in risk were limited and observed only during the historical strain and Alpha wave (period 1: OR, 1.2; 95% CI, 1.1-1.3) up to the third and last lockdown in April 2021 (period 2: OR, 1.2; 95% CI, 1.2-1.3).

Following the BA.1 wave, SARS-CoV-2 incidence fell in children during subsequent waves (BA.2 and BA.4/BA.5) (eFigures 2 and 3 in Supplement 1). Odds of infection decreased substantially for people sharing household of children of all age groups, except for children looked after by a professional in-home caregiver (eg, period 9: OR, 1.7; 95% CI, 1.6-1.8).

## Discussion

This nationwide case-control study provides a temporal perspective on the transmission of SARS-CoV-2 from children to other members of their households. It covers the emergence of more transmissible variants (notably the Delta and Omicron variants) and the introduction of vaccination for children. We found that people living with children often had a higher risk of SARS-CoV-2 infection (with highest effect sizes during the receding Delta wave in the summer of 2021, and the Omicron BA.1 wave in the winter of 2022), suggesting transmission probably from children to adults in the household. The risk associated with living with younger children (under the age of 6 years) was consistently high throughout the study, particularly for children looked after by a professional in-home caregiver. The risk associated with living with children attending primary school materialized only with the emergence of more transmissible variants, such as the Delta and Omicron variants, in a context in which the adult population was largely vaccinated; this situation was associated with the highest PAF. The risk associated with children attending middle and high school was moderate at the start of the study; it subsequently varied for children of middle-school age but declined for children of high-school age. The risk associated with children of any age decreased for members of households in the spring and summer of 2022.

Surveillance-based studies often fail to highlight the risk for people living with youngest children due to the high proportion of asymptomatic infections in those and a probable reluctance to test them in cases of exposure. This finding is consistent with previous reports of greater infectiousness in very young children than in adolescents.^19,31,32^ More frequent symptomatic infections than in older children, and closer contacts between children and with other household members, particularly parents, may account for this higher risk.^33^ The observed differences between day care centers and in-home caregivers probably reflect differences in child supervision and care.

From the summer and fall of 2021 onwards, we observed an increase in risk for the members of households including children attending primary school, and to a lesser extent, middle school. The emergence of the more transmissible Delta and Omicron variants in a population with relatively low preexisting immunity while the immunization campaign for adults was underway probably contributed to more effective circulation in these age groups and transmission to other members of their households.^14^ A similar shift was observed in England, where the proportion of index cases of individuals aged 5 to 15 years in household clusters increased strongly after the reopening of schools following the summer school holidays in 2021.^34^ Hughes et al^35^ have shown viral load was lower in children aged 5 to 18 years than in adults for the wild-type and Alpha variants, but similar for the Delta variant, potentially accounting for this increase in infectiousness over time.

Members of household including children attending middle or high school presented a moderate increase in the risk of infection, as observed in the US and England in the fall of 2020 and winter of 2021.^23,24^ The observed decrease in risk over subsequent periods, particularly for members of households including children attending high school, probably reflects the greater use of hybrid schooling methods in high schools and a high degree of vaccine coverage, particularly for the group aged 15 to 17 years. The recurrence of an increased risk for the members of households including children attending middle school during the Omicron BA.1 wave may reflect lower vaccine coverage and a waning of immunity, favoring the circulation of this variant with high immune escape capacity.

In the final months of the study, the risk decreased for the members of households including children of all age groups (except those looked after by an in-home caregiver). This probably reflects transient herd immunity in this population following the intense circulation of SARS-CoV-2 observed in the winter of 2022 (Omicron BA.1 wave).

The impact on the population of viral circulation among children depends on the increase in risk associated with exposure to children from a particular age category, and the proportion exposed to children of that age category in the population, as shown by the calculation of population-attributable fractions (Table 3). The PAFs obtained were highest for exposure to primary school-aged children during the Omicron wave. The absolute number of infections resulting from exposure to primary school-aged children during this period was increased further by the unprecedented size of the Omicron wave. Nevertheless, PAFs should be interpreted with caution here due to the inherent limitations of case-control studies, such as recruitment bias or residual confounding, for the inference of causality.

### Limitations

This study had several limitations. One major limitation of this study is the low participation rate (6.0%) among all invited cases, which resulted in the inclusion of a study population with a female preponderance, of intermediate age, and with a level of education higher than that of the overall population of individuals affected by SARS-CoV-2. The analysis was matched or adjusted for these criteria, which limits the risk of selection bias. Nonetheless, this decreases the generalizability of our results. There were probably some errors in the identification of the source case given possible multiple concurrent exposures (particularly during the periods of intense SARS-CoV-2 circulation, for instance during the Omicron BA.1 wave, ie, period 7), and child source cases were likely frequently unidentified due to high proportion of asymptomatic infections in children. Nevertheless, the findings of the descriptive analysis were consistent with those of the case-control analysis, which should not have been affected by changes in testing practices in children: both approaches revealed a similar shift in the fall of 2021, suggesting an increase in transmission from children in primary or middle school to other members of their households.

Our approach based on the measurement of risk in people living with children does not provide direct information about the role of schools in SARS-CoV-2 transmission, which is why we did not study the impact of school closures on transmission. The specific investigation of transmission in schools will require school-centered studies. Our case-control analysis is also subject to potential unmeasured confounding: parents may for instance be more likely to interact with other parents in ways that our model might have only partially accounted for, and thus be more exposed to infection, other adults acting as drivers of transmission in this hypothesis. Our descriptive analysis showing how children gradually became a prominent source of transmission suggests nonetheless that at least part of the increased risk of infection for adults sharing a household with children was driven directly by the presence of children. Our questionnaire did not include information on the number of children in the household, which could have impacted the risk of infection. Future studies will also be required to assess how the risk for people living with children evolved after the end of the study period, as immunity wanes in children.

## Conclusions

This case-control study suggests that children have contributed to the transmission of SARS-CoV-2 to other household members in France. As new generations of young children who are not yet immunized gradually join community care settings, they may continue to represent a risk of transmission to other members of their households, with a particular impact on immunocompromised adults. The vaccination of children aged between 6 months and 5 years, which was shown to reduce the risk of infection by 50.6% during the period in which the Omicron strain predominated, might have a determinant effect, decreasing this impact on the population.^36^

## Notes

Supplement 1.
eMethods.
**eTable.** Description of Cases and Controls in an Online Case-Control Study in Mainland France (October 2020 to October 2022) **eFigure 1.** Directed Acyclic Graph Representing the Causal Assumptions Underlying the Model Construction to Study the Effect of Sharing Household With Children on the Risk of SARS-CoV-2 Infection **eFigure 2.** Incidence in Children by Age Group, France, October 2020-October 2022 (New Daily Cases) **eFigure 3.** Proportion of Positive Tests by Pediatric Age Group Among All Positive Tests, France, October 2020-October 2022 **eFigure 4.** SARS-CoV-2 Daily Incidence (per 100,000) and Proportions of the Circulating Strains According to the Flash Sequencing Studies

Supplement 2.
Data Sharing Statement
