# Supplementary material for: Risk of SARS-CoV-2 Infection Among Households With Children in France, 2020-2022
Source: JAMA Netw Open. 2023 Sep 15;6(9):e2334084. doi: 10.1001/jamanetworkopen.2023.34084 (PMC10504612; doi:10.1001/jamanetworkopen.2023.34084)
Supplement: Supplement 1. — eMethods. eTable. Description of Cases and Controls in an Online Case-Control Study in Mainland France (October 2020 to October 2022) eFigure 1. Directed Acyclic Graph Representing the Causal Assumptions Underlying the Model Construction to Study the Effect of Sharing Household With Children on the Risk of SARS-CoV-2 Infection eFigure 2. Incidence in Children by Age Group, France, October 2020-October 2022 (New Daily Cases) eFigure 3. Proportion of Positive Tests by Pediatric Age Group Among All Positive Tests, France, October 2020-October 2022 eFigure 4. SARS-CoV-2 Daily Incidence (per 100,000) and Proportions of the Circulating Strains According to the Flash Sequencing Studies [file jamanetwopen-e2334084-s001.pdf]

Supplemental Online Content

Galmiche S, Charmet T, Rakover A, et al. Risk of SARS-CoV-2 infection among households with children in France, 2020-2022. *JAMA Netw Open.* 2023;6(9):e2334084. doi:10.1001/jamanetworkopen.2023.34084

eMethods.

eTable 1. Description of Cases and Controls in an Online Case-Control Study in Mainland France (October 2020 to October 2022)

eFigure 1. Directed Acyclic Graph Representing the Causal Assumptions Underlying the Model Construction to Study the Effect of Sharing Household With Children on the Risk of SARS-CoV-2 Infection

eFigure 2. Incidence in Children by Age Group, France, October 2020-October 2020 (New Daily Cases)

eFigure 3. Proportion of Positive Tests by Pediatric Age Group Among All Positive Tests, France, October 2020-October 2022

eFigure 4. SARS-CoV-2 Daily Incidence (per 100,000) and Proportions of the Circulating Strains According to the Flash Sequencing Studies

This supplemental material has been provided by the authors to give readers additional information about their work.

© 2023 Galmiche S et al. *JAMA Network Open.*

eMethods.

Study design

ComCor stands in French for "behaviours associated with the risk of SARS-CoV-2 infection".

Once weekly, the CNAM sent invitations to participate in this study by e-mail to all people testing positive in the preceding week in mainland France (the CNAM has email addresses for approximately 55% of the individuals in France covered by this insurance, who represent approximately 89% of the population of mainland France). This centralized information system was the most reliable way to include a sample of cases that was as representative as possible of the whole adult population infected by SARS-CoV-2.

The controls were enrolled via Ipsos, a market research company. This panel was the most reliable way to include a non-infected population with characteristics that we could select for matching with cases.

The school levels of the child living with the participant were daycare, child professional in-home caregiver, preschool, primary school, middle school, or high school. This grouping was chosen considering that some school-specific interventions targeted only some school levels (for instance hybrid schooling was more prevalent in high schools).

The questionnaire investigated recent activities and locations visited during the 10 days preceding the onset of symptoms or testing for asymptomatic cases (this period was decreased to 7 days after emergence of the omicron variant, which has a shorter incubation period[1]).

Statistical analysis

The sample size was mainly determined by the number of available controls who did not meet exclusion criteria (reported history of infection in the last two months). Answer was required to move forward in the questionnaire, thus we had no missing data (except for the date of last COVID-19 vaccine injection which was not required, those with missing date of last injection were categorized together).

We calculated the population attributable fraction (PAF) for living with a child by school level and by period using the *punafcc* package in Stata, which was designed for case-control studies, with the median estimate and the 2.5th and 97.5th percentiles for estimation of the 95% confidence intervals[2]. In the very few instances for which point estimates were negative, we chose not to produce an estimate rather than computing a preventive fraction, which would have had little meaning in this context.

Description of the context of the SARS-CoV-2 epidemic in France

Schools remained open according to the usual calendar during most of the study period, the major exception being the spring of 2021, when school holidays were extended, and hybrid schooling (a mixture of in-person and online teaching) was prevalent, particularly in high schools[3]. Summer holidays cover most of July and August in France.

Vaccine administration began at the end of May 2021 for 12- to 17-year-olds and in December 2021 for 5- to 11-year-olds. With grouping into school-age groups, vaccine coverage reached a plateau in February 2022 at 49% for the second dose and 4% for the third dose in the 11-14 years age group (middle school), and 72% for the second dose and 13% for the third dose in the 15-17 years age group (high school). Vaccine coverage remained very low in children aged 5-10 years (primary school) (below 10% for the second dose and close to zero for the booster). Vaccine coverage in people aged 18 or above was above 85% in February 2022 for a primary vaccine series and above 50% for the booster[4].

Description of the delimitation of the nine shorter periods used to describe changes through the study

Period 1 began on October 1, 2020, corresponding to the start of the study period and covered the second wave (historical D614G strain) and the second lockdown. Period 2 began on December 4, 2020 and covered the third wave (alpha variant) and curfew restrictions. Period 3 began on April 9, 2021 and covered the receding alpha wave and third lockdown. Period 4 began on June 14, 2021 and covered the fourth wave and the emergence of the delta variant. Period 5 began on August 14, 2021 after implementation of the sanitary pass (proof of vaccine or past episode of infection, or recent negative SARS-CoV-2 test to enter a series of public places) and covered

the receding fourth wave (delta variant). Period 6 began on October 2, 2021 and covered the start of the fifth wave (delta variant). Period 7 began on December 20, 2021 and covered the fifth wave, during which the omicron variant (BA.1 subvariant) predominated. Period 8 began on March 18, 2022 (following the lifting of obligation to wear a mask in schools and most indoor spaces) and covered the omicron BA.2 wave. Period 9 began on May 19, 2022 (following the lifting of the obligation to wear a mask on public transport) and covered the omicron BA.4/BA.5 wave, extending to the end of the study period on October 2, 2022.

We took the incubation period into account, by applying a four- or five-day lag period (depending on the predominant strain in circulation at the time) between the changes in restrictions and the start of the next period.

References

1. Galmiche S, Cortier T, Charmet T, et al. SARS-CoV-2 incubation period across variants of concern, individual factors, and circumstances of infection in France: a case series analysis from the ComCor study. *The Lancet Microbe*. 2023;4(6):e409-e417. doi:10.1016/S2666-5247(23)00005-8

2. Newson RB. Attributable and Unattributable Risks and Fractions and other Scenario Comparisons. *The Stata Journal*. 2013;13(4):672-698. doi:10.1177/1536867X1301300402

3. Ministère de l'Education Nationale et de la Jeunesse. Préparer son plan de continuité pédagogique. éduscol | Ministère de l'Éducation nationale et de la Jeunesse - Direction générale de l'enseignement scolaire. Accessed December 1, 2022. https://eduscol.education.fr/2227/preparer-son-plan-de-continuite-pedagogique

4. Données relatives aux personnes vaccinées contre la Covid-19 (VAC-SI) - data.gouv.fr. Accessed June 21, 2023. https://www.data.gouv.fr/fr/datasets/donnees-relatives-aux-personnes-vaccinees-contre-la-covid-19-1/

© 2023 Galmiche S et al. *JAMA Network Open.*

eTable 1. Description of cases and controls in an online case-control study in mainland France (October 2020 to October 2022)

	Cases	Controls	*p*-values[a]	Periods[b]
	175,688	43,922		
**Comorbidities**				
Immunosuppressive treatment				
No immunosuppressor treatment	61,089/63,284 (96.5%)	15,286/15,821 (96.6%)	<0.001	1 to 6
Immunosuppressive treatment	1,685/63,284 (2.7%)	465/15,821 (2.9%)		
Did not reply	436/63,284 (0.7%)	69/15,821 (0.4%)		
Any immune suppression				
No immune suppression	63,952/68,268 (93.7%)	16,135/17,067 (94.5%)	<0.001	8 to 9
Immune suppression	3,545/68,268 (5.2%)	822/17,067 (4.8%)		
Did not reply	772/68,268 (1.1%)	110/17,067 (0.6%)		
**Smoking status**				
No smoking nor nicotine consumption	139,810 (79.6%)	33,284 (75.8%)	<0.001	
Smoker with or without nicotine consumption	27,072 (15.4%)	8,644 (19.7%)		
Nicotine consumption alone	8,807 (5.0%)	1,994 (4.5%)		
**COVID-19 vaccine status**				
Not vaccinated	42,665 (24.3%)	11,676 (26.6%)	<0.001	
1 dose <90 days	3,291 (1.9%)	870 (2.0%)		
1 dose 90-179 days	553 (0.3%)	260 (0.6%)		
1 dose ≥180 days	619 (0.4%)	264 (0.6%)		
2 doses <90 days	7,305 (4.2%)	3,024 (6.9%)		
2 doses 90-179 days	13,717 (7.8%)	3,150 (7.2%)		
2 doses ≥180 days	7,252 (4.1%)	2,091 (4.8%)		
3 doses <90 days	26,331 (15.0%)	6,794 (15.5%)		
3 doses 90-179 days	40,054 (22.8%)	6,810 (15.5%)		
3 doses ≥180 days	16,703 (9.5%)	2,925 (6.7%)		
4 doses <90 days	2,726 (1.6%)	560 (1.3%)		
Undated last dose of vaccine	7,161 (4.1%)	3,669 (8.4%)		
**Professional category …**				
… of the reference person in the household				
Employee	4,397/26,944 (16.3%)	1,587/6,736 (23.6%)	<0.001	1 to 2
Intermediate profession	5,908/26,944 (21.9%)	1,452/6,736 (21.6%)		1 to 2
Independent profession	1,296/26,944 (4.8%)	241/6,736 (3.6%)		1 to 2
Senior executive	8,623/26,944 (32.0%)	1,618/6,736 (24.0%)		1 to 2
Worker	2,425/26,944 (9.0%)	523/6,736 (7.8%)		1 to 2
Retired	3,225/26,944 (12.0%)	906/6,736 (13.5%)		1 to 2
Unemployed or inactive people	1,071/26,944 (4.0%)	409/6,736 (6.1%)		1 to 2
… of the participant				
Employee	26,754/148,744 (18.0%)	8,794/37,186 (23.6%)	<0.001	3 to 9
Senior executive	45,919/148,744 (30.9%)	6,966/37,186 (18.7%)		3 to 9
Intermediate profession	29,304/148,744 (19.7%)	6,877/37,186 (18.5%)		3 to 9
Worker, farmer, independent profession	10,413/148,744 (7.0%)	3,382/37,186 (9.1%)		3 to 9
Retired	26,520/148,744 (17.8%)	7,175/37,186 (19.3%)		3 to 9
Unemployed or inactive people	5,619/148,744 (3.8%)	2,813/37,186 (7.6%)		3 to 9
Students	4,215/148,744 (2.8%)	1,180/37,186 (3.2%)		3 to 9
**Number of people in the household**				
1	34,687 (19.7%)	9,096 (20.7%)	<0.001	
2	58,982 (33.6%)	15,186 (34.6%)		
3	32,951 (18.8%)	8,451 (19.2%)		
4	34,481 (19.6%)	7,897 (18.0%)		

© 2023 Galmiche S et al. *JAMA Network Open.*

	Cases	Controls	*p*-values[a]	Periods[b]
	175,688	43,922		
5	10,946 (6.2%)	2,553 (5.8%)		
≥ 6	3,641 (2.1%)	739 (1.7%)		
**Work in the exposure period**				
No work	55,168 (31.4%)	16,009 (36.4%)	<0.001	
Non-office work	51,275 (29.2%)	11,996 (27.3%)		
Partially remote office work	26,269 (15.0%)	6,296 (14.3%)		
Completely remote office work	11,308 (6.4%)	2,320 (5.3%)		
On-site office work	31,668 (18.0%)	7,301 (16.6%)		
**Attendance of retail facility**				
Any retail facility	5,864/7,308 (80.2%)	1,498/1,827 (82.0%)	0.456	1
Supermarket	82,490/168,380 (49.0%)	26,702/42,095 (63.4%)	<0.001	2 to 9
Convenience store	58,873/168,380 (35.0%)	15,056/42,095 (35.8%)	0.013	2 to 9
Shopping mall	26,227/168,380 (15.6%)	9,433/42,095 (22.4%)	<0.001	2 to 9
Market	19,343/168,380 (11.5%)	5,897/42,095 (14.0%)	<0.001	2 to 9
Other retail facility	5,635/168,380 (3.3%)	2,012/42,095 (4.8%)	<0.001	2 to 9
**Public short-distance transport**				
Bus	19,285 (11.0%)	6,584 (15.0%)	<0.001	
Metro	23,348 (13.3%)	5,847 (13.3%)	0.907	
Train	10,140 (5.8%)	2,992 (6.8%)	<0.001	
Tram	10,184 (5.8%)	3,493 (8.0%)	<0.001	
**Long-distance travel**				
Travel outside region of residence	37,907 (21.6%)	9,804 (22.3%)	0.003	
Travel abroad	8,976 (5.1%)	1,779 (4.1%)	<0.001	
Train travel	9,087 (5.2%)	1,752 (4.0%)	<0.001	
Airplane travel	6,329 (3.6%)	1,091 (2.5%)	<0.001	
Bus travel	2,749 (1.6%)	642 (1.5%)	0.12	
**Car-pooling**				
Any carpooling	4,341/35,952 (12.1%)	928/8,988 (10.3%)	<0.001	1 to 3
With family and friends	29,620/139,736 (21.2%)	7,350/34,934 (21.0%)	0.567	4 to 9
Via car sharing plateform	1,355/139,736 (1.0%)	692/34,934 (2.0%)	<0.001	4 to 9
**Gatherings**				
Private gathering	78,974 (45.0%)	21,405 (48.7%)	<0.001	
Professional meeting	47,777 (27.2%)	10,303 (23.5%)	<0.001	
Classes	10,498 (6.0%)	2,840 (6.5%)	<0.001	
Cultural gathering	26,003 (14.8%)	6,224 (14.2%)	0.002	
Religious gathering	6,510 (3.7%)	1,830 (4.2%)	<0.001	
**Sports**				
Swimming pool	8,680 (4.9%)	2,529 (5.8%)	<0.001	
Indoor sports	12,235 (7.0%)	3,060 (7.0%)	0.984	
**Leisure activities**				
Bar or restaurant	1,900/7,308 (26.0%)	286/1,827 (15.7%)	<0.001	1
Restaurant	51,212/168,380 (30.4%)	13,885/42,095 (33.0%)	<0.001	2 to 9
Bar	22,802/168,380 (13.5%)	6,156/42,095 (14.6%)	<0.001	2 to 9
Party	2,380/47,216 (5.0%)	323/11,804 (2.7%)	<0.001	1 to 4
Party in a private setting	12,486/128,472 (9.7%)	4,344/32,118 (13.5%)	<0.001	5 to 9
Night-club	3,558/128,472 (2.8%)	727/32,118 (2.3%)	<0.001	5 to 9

Legend: a: *p*-values: Chi-squared test; b: Periods: indicated when the variable was collected and introduced in the model in some of the periods only (denominator is indicated); blank means that the variable remained unchanged through the whole study period (denominator is the complete population of cases or controls). Numbers are sums over the nine periods of the study of the averages of the 100 databases generated for each period using a bootstrapping procedure.

© 2023 Galmiche S et al. *JAMA Network Open.*

eFigure 1. Directed acyclic graph representing the causal assumptions underlying the model construction to study the effect of sharing household with children on the risk of SARS-CoV-2 infection

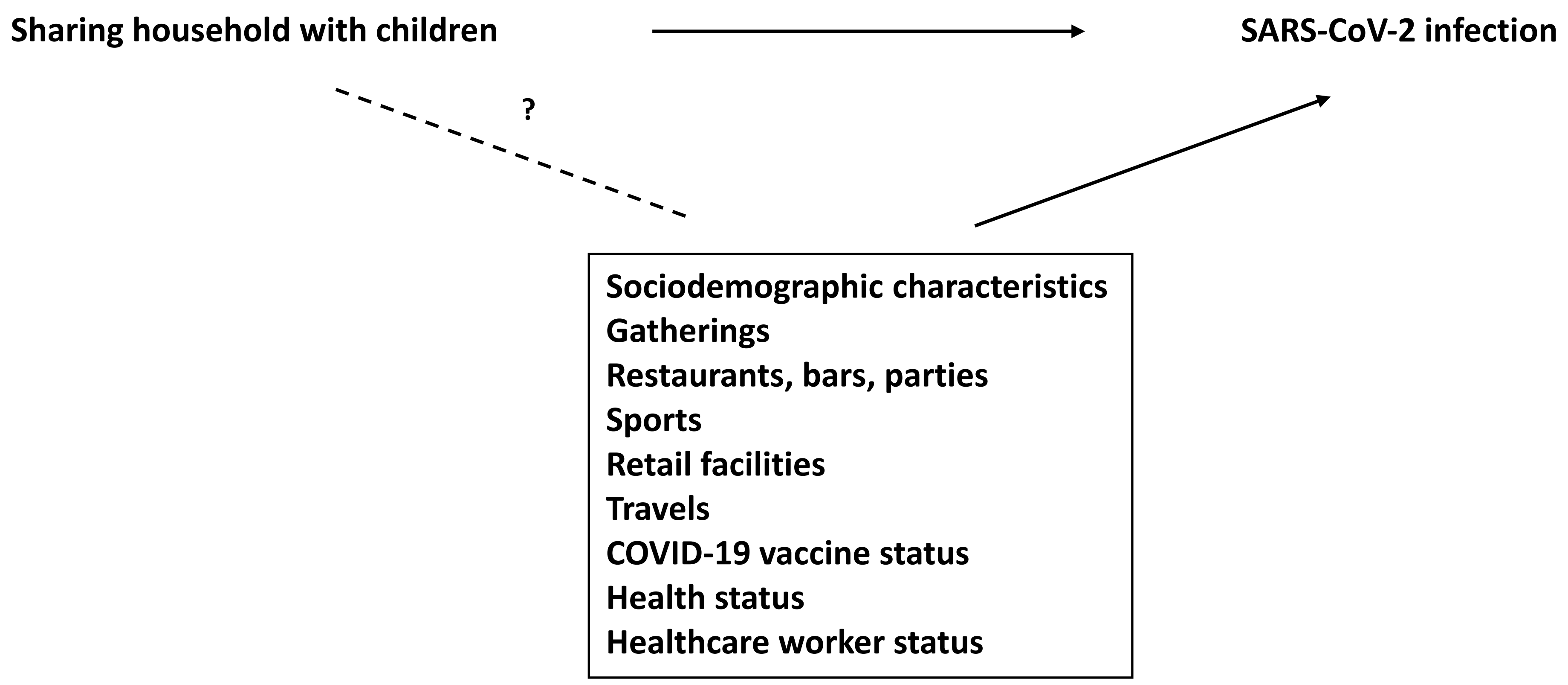

© 2023 Galmiche S et al. *JAMA Network Open.*

eFigure 2. Incidence in children by age group, France, October 2020-October 2020 (new daily cases) (data provided by the French government)

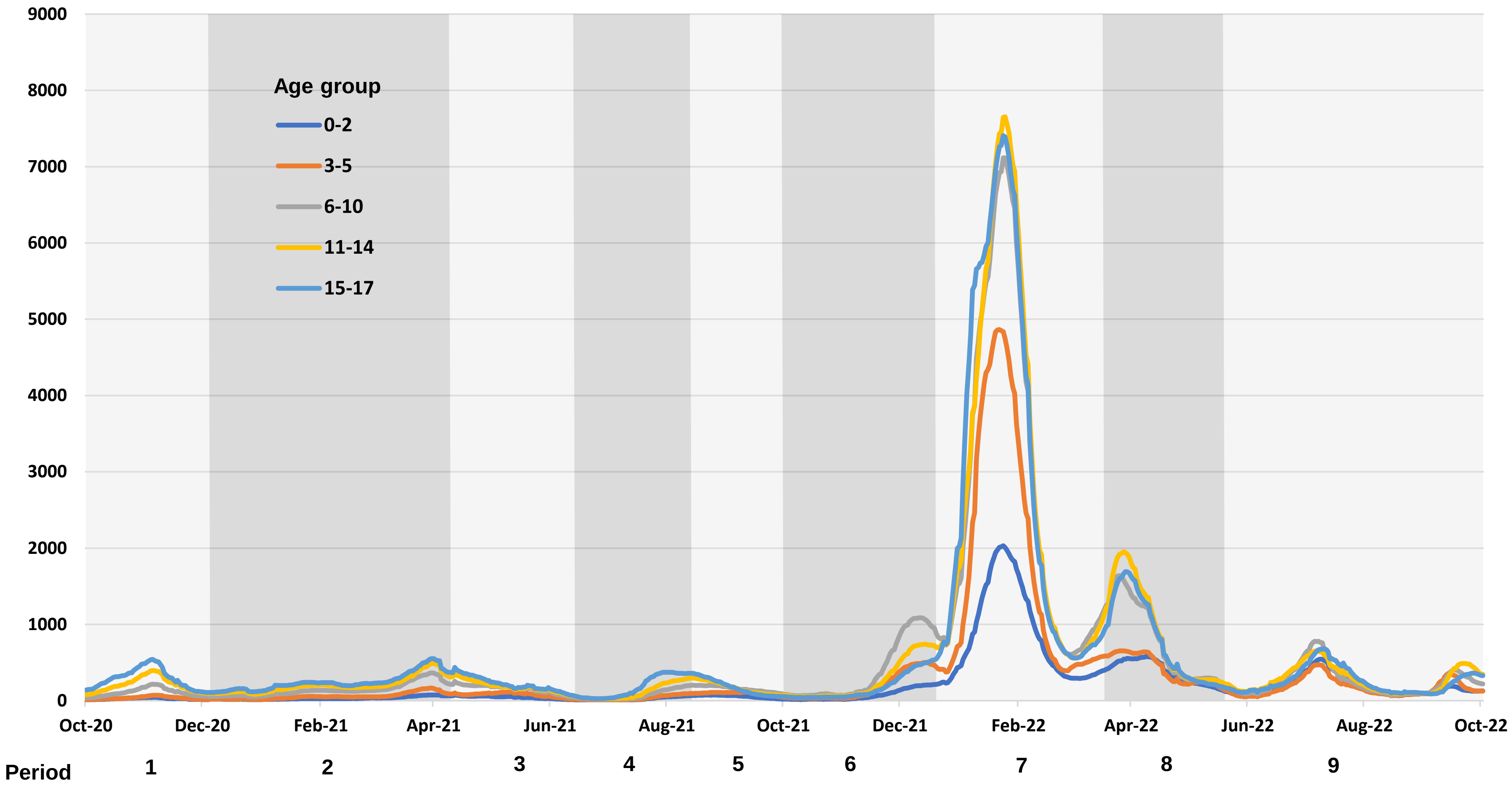

Legend: Study conducted in France between October 2020 and October 2022. The data on positive SARS-CoV-2 tests are public data provided by the French government. The areas shaded in gray indicate the 9 periods into which the study period was divided. Data provided by the French government.

© 2023 Galmiche S et al. *JAMA Network Open.*

eFigure 3. Proportion of positive tests by pediatric age group among all positive tests, France, October 2020-October 2022 (%)

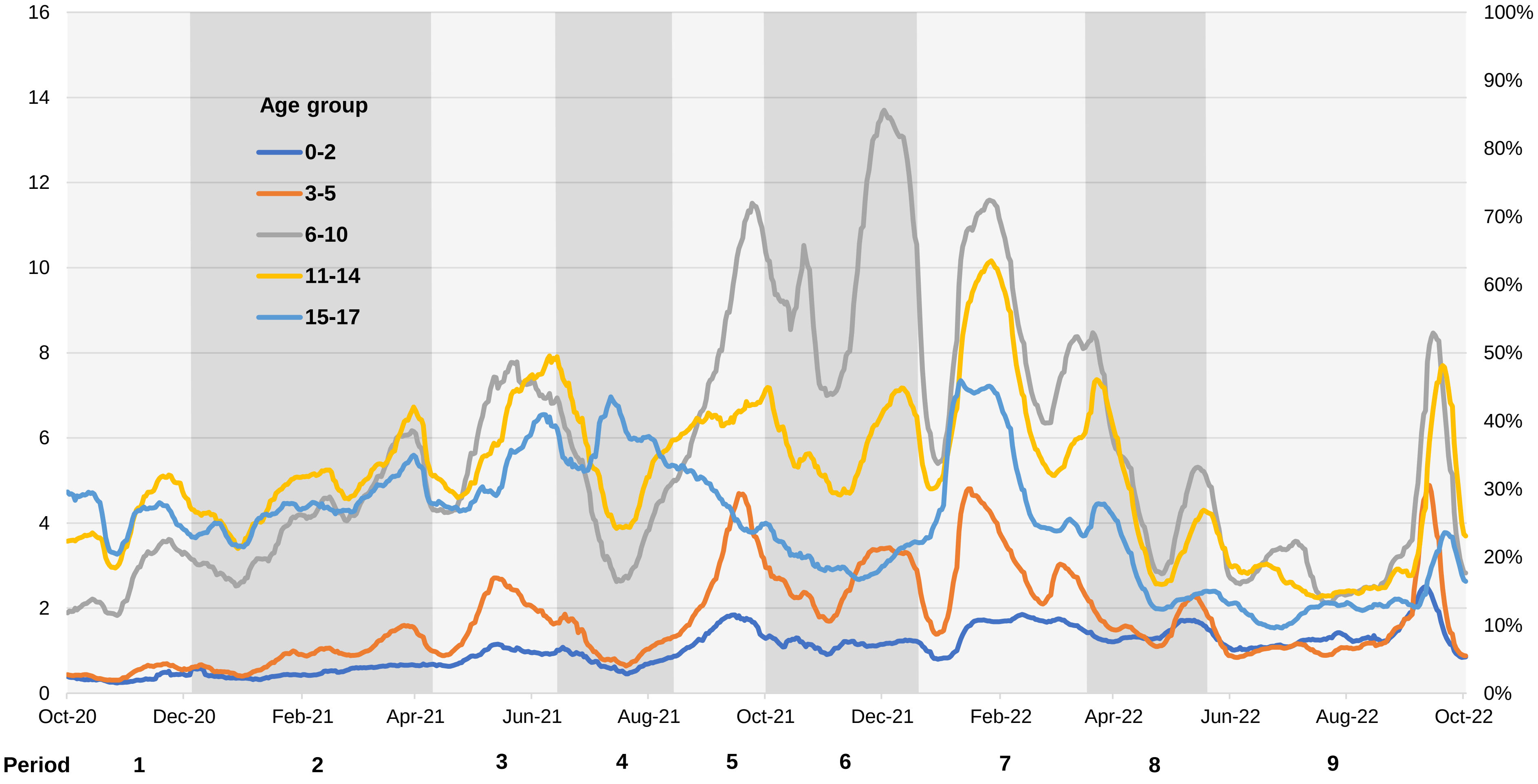

Legend: Study conducted in France between October 2020 and October 2022. The data on positive SARS-CoV-2 tests are public data provided by the French government. The areas shaded in gray indicate the 9 periods into which the study period was divided. Data provided by French government.

© 2023 Galmiche S et al. *JAMA Network Open.*

eFigure 4. SARS-CoV-2 daily incidence (per 100,000) and proportions of the circulating strains according to the Flash sequencing studies

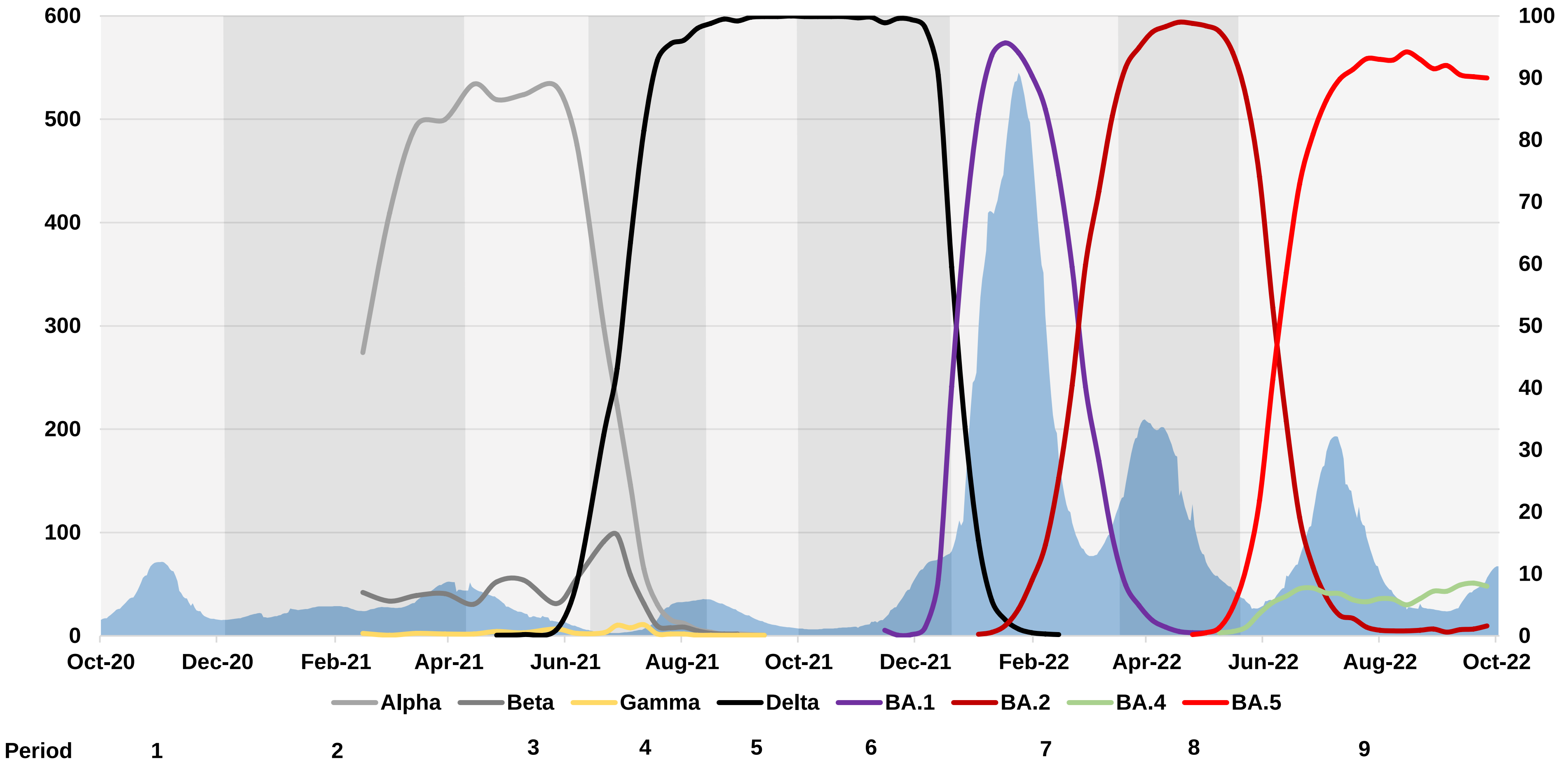

Legend: The Flash sequencing studies were conducted weekly on a random sample of SARS-CoV-2 tests. The periods were defined on the basis of changes in SARS-CoV-2 incidence, predominant circulating strain, and major non-pharmaceutical interventions (the periods are indicated by shaded gray areas).

© 2023 Galmiche S et al. *JAMA Network Open.*
