# Supplementary material for: Risk of SARS-CoV-2 Infection Among Households With Children in France, 2020-2022
Source: JAMA Netw Open. 2023 Sep 15;6(9):e2334084. doi: 10.1001/jamanetworkopen.2023.34084 (PMC10504612; doi:10.1001/jamanetworkopen.2023.34084)
Supplement: Supplement 2. — Data Sharing Statement [file jamanetwopen-e2334084-s002.pdf]

Data Sharing Statement

Galmiche. Risk of SARS-CoV-2 Infection Among Households With Children in France, 2020-2022. *JAMA Netw Open*. Published September 15, 2023.
doi:10.1001/jamanetworkopen.2023.34084

Data
Data available: No

Additional Information
Explanation for why data not available: The data that support the findings of this study are available from Institut Pasteur. Restrictions apply to the availability of these data, which were used under authorized agreement for this study from the data protection authority, the Commission Nationale de l'Informatique et des Libertés (CNIL). Access to these pseudonymized data would therefore require prior authorization by the CNIL.
